# Identification of key genes associated with perineural invasion in stage II colorectal cancer and their prognostic implications

**DOI:** 10.3389/fonc.2025.1612143

**Published:** 2025-08-27

**Authors:** Chen Chang, Bin Zhang, Jingli Chen, Guorong Wang

**Affiliations:** ^1^ Department of Pathology, Shaanxi Provincial People’s Hospital, Xi’an, Shaanxi, China; ^2^ FirstDepartment of General Surgery, Shaanxi Provincial People’s Hospital, Xi’an, Shaanxi, China

**Keywords:** colorectal cancer, perineural invasion, differentially expressed genes, nomogram model, prognostic evaluation

## Abstract

**Objective:**

This study aimed to identify key genes associated with perineural invasion (PNI) in stage II colorectal cancer (CRC) and develop a prognostic nomogram. The goal was to create a model for more precise prognosis assessment and to guide personalized treatment for stage II CRC patients with PNI.

**Methods:**

Bioinformatic analysis of The Cancer Genome Atlas (TCGA) database was used to identify differentially expressed genes (DEGs) associated with PNI in stage II CRC. Kaplan-Meier and Cox regression analyses identified prognostic genes for overall survival (OS). These genes, along with clinical factors, were integrated into a nomogram. The model’s performance was evaluated using calibration curves, receiver operating characteristic (ROC)/area under the curve (AUC) analysis, and decision curve analysis (DCA). Key gene expression in CRC tissues was validated by immunohistochemistry (IHC) and correlated with clinicopathological parameters.

**Results:**

We identified 33 DEGs associated with stage II CRC and PNI. High expression of CLDN18 and FTCD were independent poor prognostic indicators. A nomogram incorporating these genes and clinical factors accurately predicted 1-, 3-, and 5-year overall survival (OS), with AUC values exceeding 0.7. Calibration curves and DCA confirmed the model’s clinical utility. Immunohistochemistry (IHC) revealed that Claudin 18 protein expression was significantly higher in PNI-positive CRC tissues (P < 0.05) and correlated with age and lymphatic invasion (P < 0.05).

**Conclusion:**

We developed a novel prognostic nomogram for stage II CRC patients with PNI. This model provides a new tool for CRC prognosis, deepens the understanding of PNI pathogenesis, and helps identify therapeutic targets like Claudin 18, whose expression was confirmed as a potential biomarker. This tool can enhance personalized treatment strategies for this high-risk patient population.

## Introduction

Colorectal cancer is the third most prevalent malignancy worldwide and has the third highest associated mortality rate (1). In China, the incidence rate of CRC is the third highest among malignant tumors at 28.2 per 100,000, while the mortality rate is fifth at 13.61 per 100,000 (2). Currently, the Tumor-Node-Metastasis (TNM) classification system is the standard for predicting survival and recurrence in colorectal cancer patients. In addition to TNM staging, several supplementary risk factors can be applied to further stratify risks (3–5). The inclusion of these additional stratification factors indicates a potential need to refine and enhance the TNM system. Numerous studies have demonstrated a strong correlation between PNI and decreased survival rates as well as increased recurrence rates in CRC. The National Comprehensive Cancer Network (NCCN) clinical practice guidelines classify PNI as a high-risk feature for CRC recurrence, recommending adjuvant therapy for stage II CRC patients with positive PNI findings (4). However, the current guidelines focus mainly on the significance of PNI in stage II CRC, leaving its prognostic value in other stages ambiguous (6, 7).

Perineural invasion (PNI) refers to the way tumors invade by infiltrating cancer cells along nerve fibers, which is different from direct extension and vascular metastasis. This phenomenon was first identified in malignant tumors of the head and neck region (8). The average detection rate of PNI using hematoxylin and eosin (HE) staining is about 17%, with a range of 8% to 42%. In contrast, immunohistochemical methods can achieve detection rates of up to 70% for PNI-related protein expression (9). Recent studies on PNI have shown significant links between PNI and factors such as poor differentiation, T staging, and lymphatic metastasis. Thus, PNI may serve as an alternative biomarker for the aggressive phenotype of colorectal cancer (10).

Currently, there are no studies that explore prognostic stratification methods using molecular expressions related to PNI in stage II CRC. This study will develop a nomogram model that incorporates PNI-related molecular expressions to predict the prognosis for patients with stage II CRC. By doing so, this approach aims to provide more comprehensive references for assessing the prognosis of colorectal cancer patients.

## Materials and methods

### Data acquisition

We acquired and processed data from the TCGA database (https://portal.gdc.cancer.gov), specifically focusing on the TCGA-COADREAD project related to colorectal cancer. RNA sequencing (RNA-seq) data were analyzed using the STAR pipeline to extract FPKM-format data for stage II colorectal cancer. This data was then matched with the corresponding clinical information.

### Study population and clinical data collection

This was a retrospective study using archived pathological tissue samples from 118 patients who had undergone radical resection for colorectal cancer at Shaanxi Provincial People’s Hospital between January 2017 and December 2018. All samples were residual tissues obtained after the completion of clinical diagnosis. The inclusion criteria were as follows: 1) postoperative histopathological diagnosis confirming colorectal cancer; 2) no preoperative radiotherapy, chemotherapy, or other systemic tumor-targeted therapy; and 3) no history of other malignant tumors. The study protocol was approved by the Ethics Committee of Shaanxi Provincial People’s Hospital (Approval No. 2023-R057), which granted a waiver for individual patient consent due to the retrospective nature of the research and the use of fully anonymized patient data.

We conducted a comprehensive collection and documentation of all patients’ clinical and pathological characteristics. This included various parameters, such as gender, age at diagnosis, tumor location, and clinical staging. For TNM staging, we evaluated the size of the primary tumor and depth of invasion (T), the status of regional lymph node metastasis (N), and the presence of distant metastasis (M). We also classified histological grading as well-differentiated, moderately differentiated, or poorly differentiated.

### Immunohistochemical staining analysis

We used the PV8000 immunohistochemistry kit and the DAB staining kit (Zhongshan Jinqiao Biotechnology) for immunohistochemical detection. The primary antibody was Rabbit anti-Claudin 18 polyclonal antibody (21126-1-AP, Proteintech), and experiments followed the manufacturer’s protocol. The specific procedural steps included the following: Firstly, tissue sections were subjected to deparaffinization and rehydration. Next, we blocked endogenous peroxidase activity using 3% hydrogen peroxide (H_2_O_2_). Following this, we performed microwave-mediated antigen retrieval with a citrate buffer. After antigen retrieval, tissue sections were incubated overnight at 4°C with the Claudin 18 antibody, followed by DAB staining to visualize the immunoreactivity. Subsequently, sections were counterstained with hematoxylin and dehydrated. Finally, the specimens were mounted using neutral resin. Immunohistochemical reactions resulting in brown-yellow granules were considered positive. We quantified the relative expression levels of the proteins based on staining intensity and the percentage of positively stained area. The scoring criteria for staining intensity were as follows: no staining (0 points), weak staining (1 point), moderate staining (2 points), and strong staining (3 points). The scoring criteria for the stained area were: ≤10% staining (0 points), 11%-20% (1 point), 21%-50% (2 points), 51%-70% (3 points), and >70% (4 points). The final score was the product of the two scores. A total score of ≥6 indicated high expression, while a score of <6 indicated low expression. All IHC procedures adhered to strict quality control standards, and the results were independently evaluated by two experienced pathologists to ensure accuracy and reproducibility of the findings.

### Differential expression gene analysis

We used the DESeq2 package in R to perform differential expression analysis on the raw counts matrix. This analysis compared stage II CRC with other stages and examined tumors with and without PNI. We followed standard protocols for the analysis, including data normalization using the Variance Stabilizing Transformations (VST) method from DESeq2. We applied stringent filtering criteria, requiring a log2 fold change (FC)| > 1 and a p < 0.05 to identify differentially expressed genes (DEGs) between the two groups. Subsequent analyses examined both unique and shared DEGs across the different groups. We used the ggplot2 and VennDiagram packages to create clear graphical representations of the results.

### Survival analysis and nomogram model construction

The Surv cutpoint function was utilized to calculate Kaplan-Meier curves for overall survival (OS), with differences assessed using the log-rank test. Genes associated with overall survival in stage II colorectal cancer were identified, and those with p>0.05 were excluded to obtain a set of prognostic-related genes. Univariate and multivariate Cox regression analyses were performed to determine the independent prognostic factors. A nomogram was constructed based on these prognostic factors to provide a comprehensive assessment of patient survival probabilities. The effectiveness of the nomogram model was evaluated through receiver operating characteristic (ROC) curves, area under the curve (AUC) values, calibration curves, and decision curve analysis (DCA).

To test the proportional hazards assumption and fit survival regression, the survival package was employed, and the results were visualized using the survminer and ggplot2 packages. During the univariate analysis, the optimal cut-off values were determined using the surv_cutpoint function from the survminer package. The R package “rms” was utilized to generate Cox regression models, the nomogram, and calibration plots. Survival probabilities were predicted using the R package “pec.” ROC curves and DCA curves were plotted using the R packages “timeROC” and “ggDCA,” respectively. Statistical significance was defined as a p-value of less than 0.05.

### Data analysis

Statistical analysis of the collected data was performed using software such as Microsoft Excel, GraphPad Prism 7.0, and SPSS 23.0. This study used the chi-square test to evaluate the significance of differences between the two groups based on contingency tables. Additionally, we used the χ² test and Fisher’s exact test to analyze correlations among clinical pathological parameters. All tests were performed using a two-sided approach with a significance level set at α=0.05.

## Results

### Differential gene screening associated with PIN in stage II colorectal cancer

**Figure 1:**
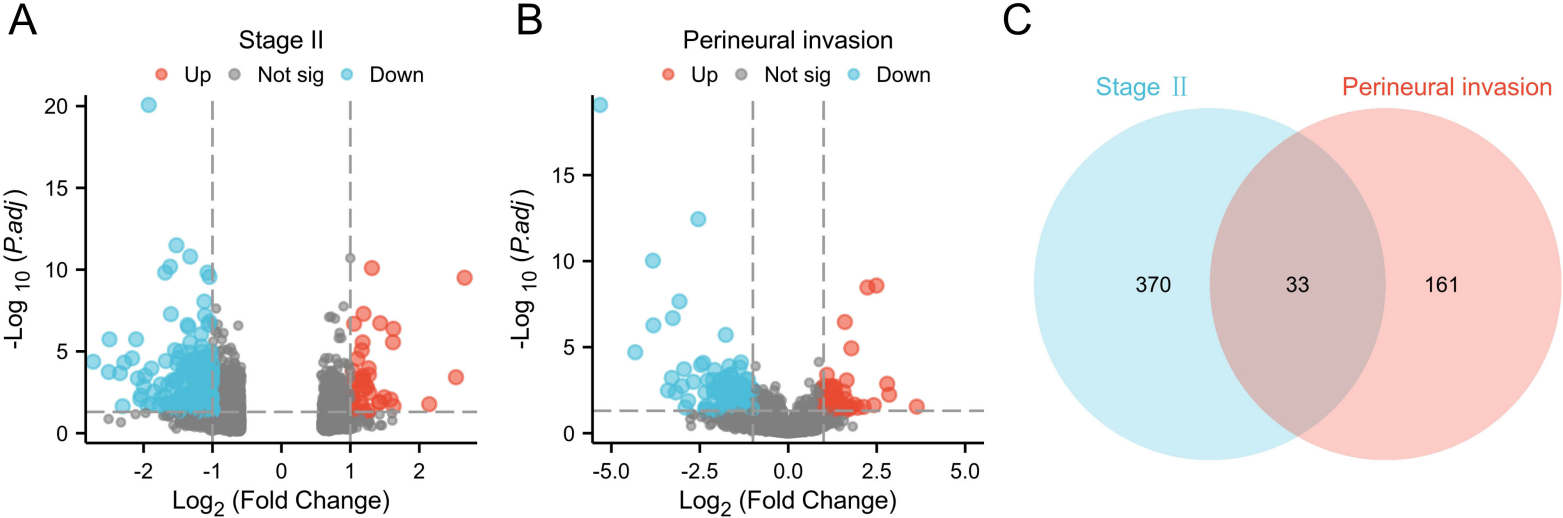
Differential gene screening. **(A)** Volcano plot of differential analysis for stage II CRC from the TCGA-COADREAD database; **(B)** Volcano plot of differential analysis related to PNI in CRC; **(C)** Venn diagram of differentially expressed genes.

We screened differential genes from the TCGA database using thresholds of log2 fold change (FC) | > 1 and p < 0.05. This process identified 370 DEGs related to stage II CRC and 161 DEGs associated with PNI. The results were visualized with a volcano plot, which clearly displayed both upregulated and downregulated genes. Next, we conducted a Venn diagram analysis to identify unique and shared genes between the two groups, revealing 33 commonly expressed genes that were significantly altered (**Figure 1**).

### Identification of prognostic-related genes

**Figure 2:**
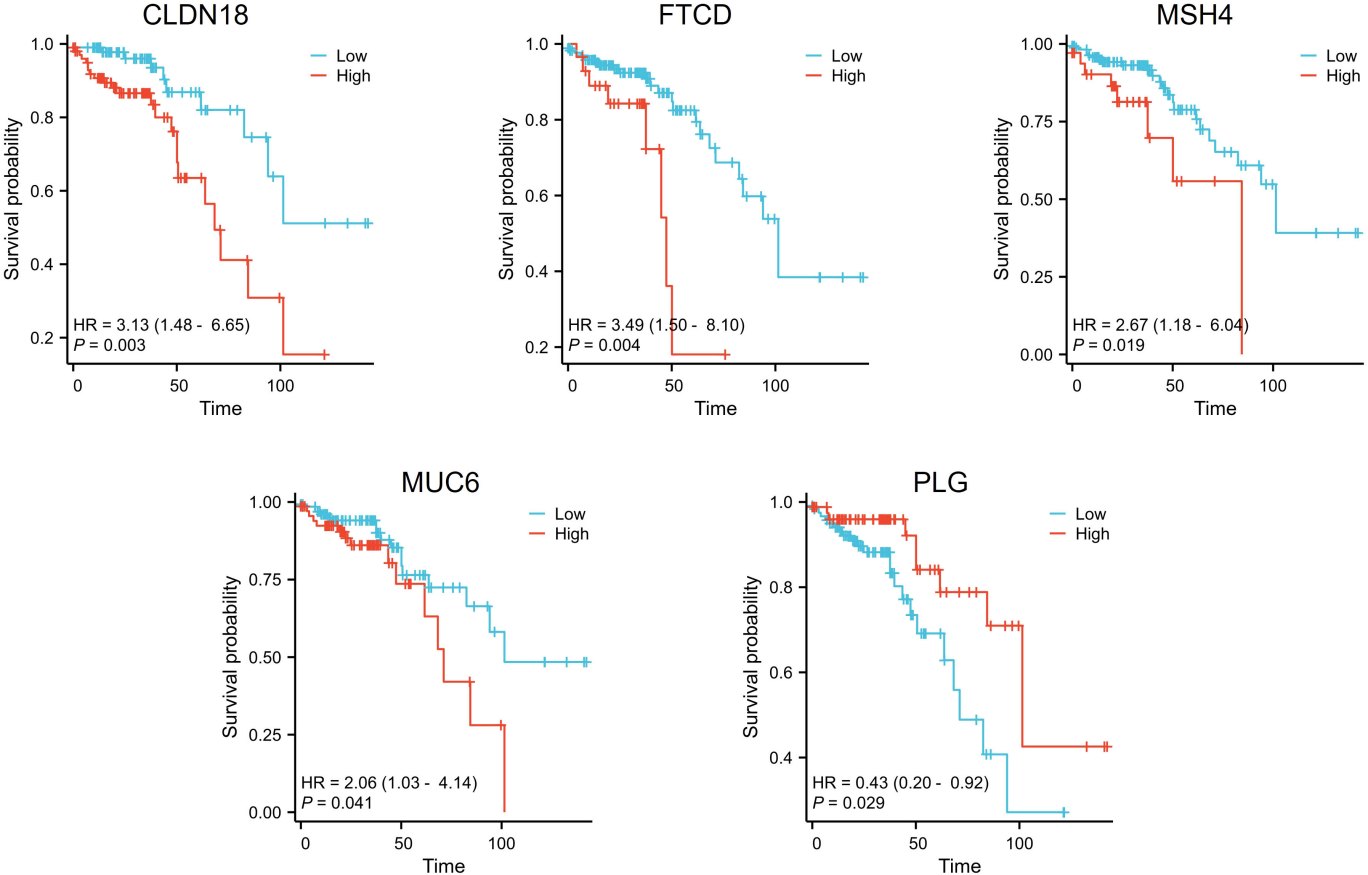
Survival analysis of differential genes in stage II colorectal cancer patients. Patients with high expression of CLDN18, FTCD, MSH4, and MUC6 displayed shorter overall survival times, while those with high expression of PLG exhibited longer overall survival times.

We downloaded and organized RNA sequencing data and corresponding clinical information from the TCGA-COADREAD project. The clinical data for 208 patients with stage II CRC were complete and used in the survival analysis. We applied a threshold of p < 0.05 to identify five genes significantly associated with overall survival: CLDN18, FTCD, MSH4, MUC6, and PLG. Among the identified genes, high expression of PLG was associated with longer overall survival. In contrast, higher expression of the other genes (CLDN18, FTCD, MSH4, and MUC6) correlated with shorter overall survival (**Figure 2**).

**Figure 3:**
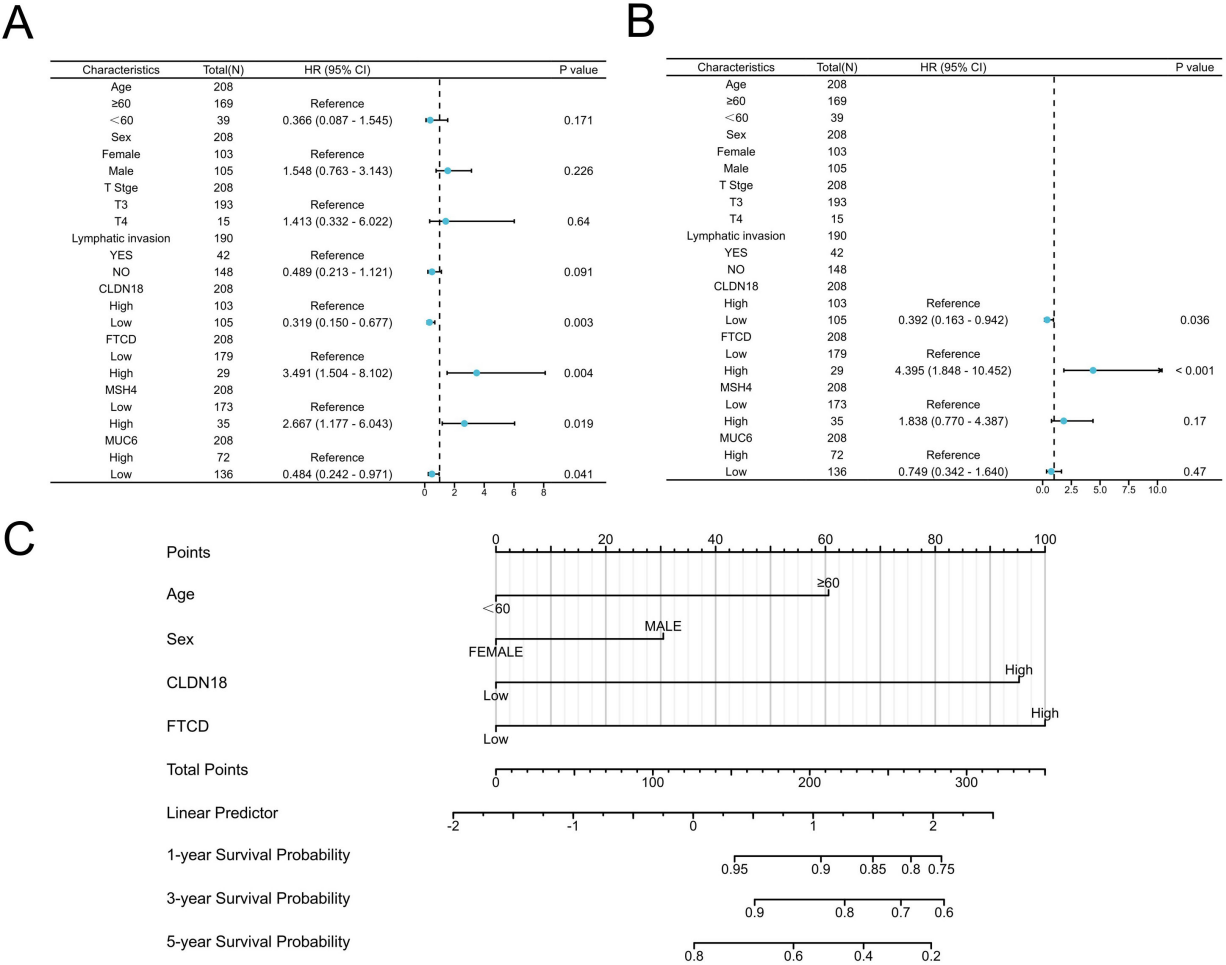
Establishment of prognostic model based on COX regression analysis. **(A, B)** Univariate and multivariate COX regression analyses; **(C)** Construction of the prognostic Nomogram model for Stage II colorectal cancer based on COX analysis results.

Among the five prognostic genes identified from the Kaplan-Meier analysis, we first excluded PLG, as our study aimed to build a risk model based on adverse prognostic factors (oncogenes), whereas high expression of PLG was associated with better survival and thus considered a protective factor. For the remaining four oncogenes (CLDN18, FTCD, MSH4, and MUC6), we proceeded with univariate Cox regression analysis. To select the most robust candidates for the subsequent multivariate analysis and nomogram construction, we applied a stricter significance threshold for inclusion (P < 0.01). As shown in the univariate analysis (**Figure 3A**), only CLDN18 (P = 0.003) and FTCD (P = 0.004) met this criterion, while MSH4 (P = 0.019) and MUC6 (P = 0.041) did not. Therefore, only CLDN18 and FTCD were carried forward into the final model for multivariate analysis, which confirmed them as independent adverse prognostic factors (**Figure 3B**).

### Construction of prognostic model for stage II colorectal cancer patients

Given that predicting the prognosis of CRC patients using a single biomarker or clinical characteristic is often difficult, we developed a comprehensive prognostic nomogram model that integrates CLDN18 and FTCD with clinical factors like age and gender. This nomogram is designed to assess the probability of OS in CRC patients. In the nomogram, the length of each variable’s line indicates its contribution to the prognosis. The analysis showed that CLDN18 and FTCD significantly impacted the OS of patients with stage II CRC compared to other parameters (**Figure 3C**).

### Validation of the prognostic capability of the nomogram model

**Figure 4:**
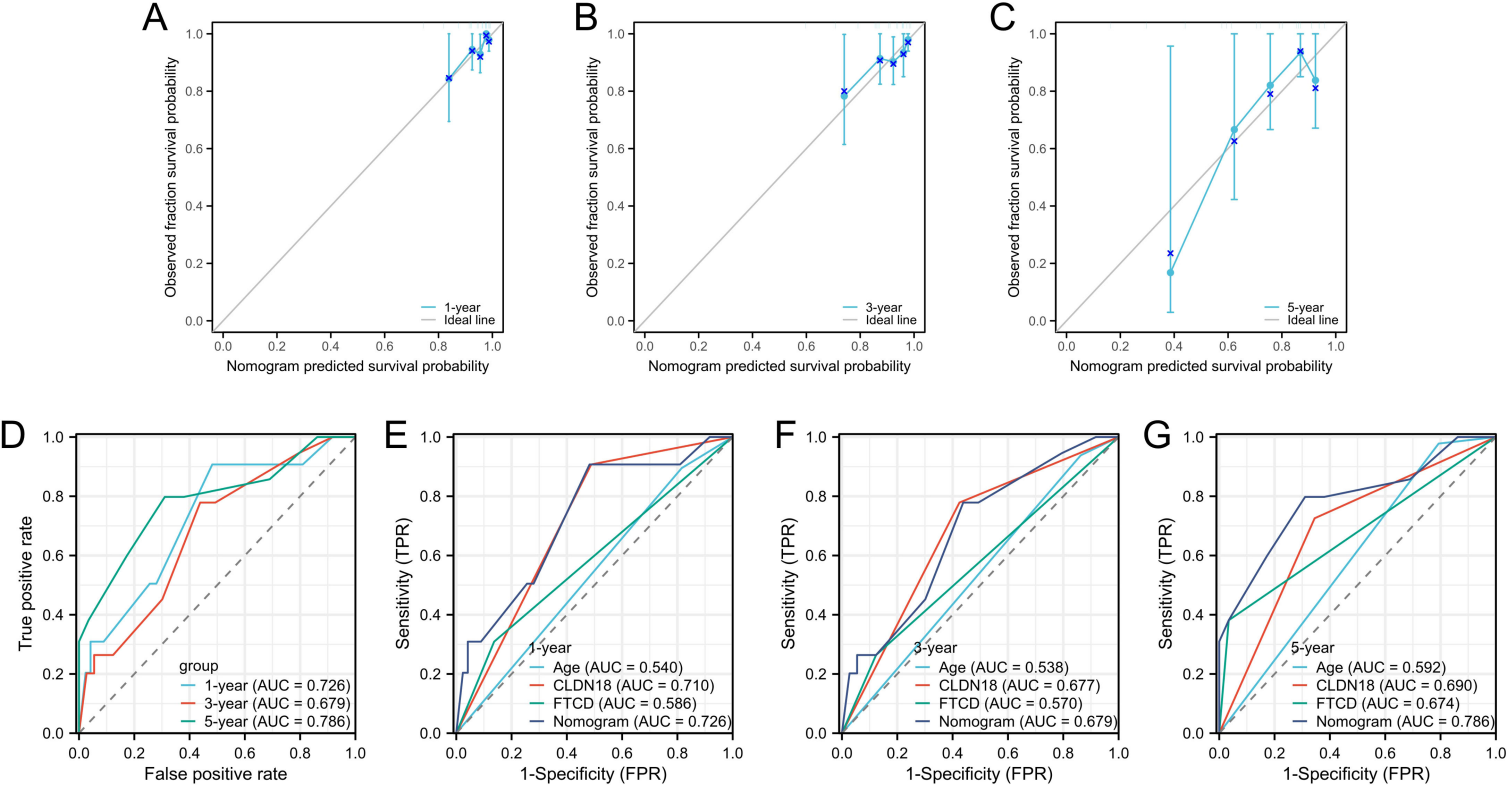
Validation of prognostic capability of the nomogram model. **(A–C)** Calibration plots for prognostic predictions at 1-year, 3-year, and 5-year intervals; **(D)** ROC curves for overall survival rates at 1 year, 3 years, and 5 years; **(E–G)** ROC curves for the Nomogram model and CLDN18 and FTCD at 1-year, 3-year, and 5-year intervals.

We evaluated the prognostic model’s performance using calibration curves, AUC values, and DCA. First, we plotted calibration curves for the nomogram. These curves showed that the model’s predicted probabilities of overall survival at 1, 3, and 5 years closely matched the actual outcomes for stage II colorectal cancer patients, indicating high accuracy (**Figures 4A–C**). Next, we generated ROC curves to assess the predictive sensitivity and specificity of the nomogram model. The AUC values for overall survival were 0.726 at 1 year, 0.679 at 3 years, and 0.786 at 5 years (**Figure 4D**). Moreover, the AUC values for the nomogram model in predicting 1-year, 3-year, and 5-year survival were higher than those for age, CLDN18, and FTCD. This highlights the superior predictive performance of the nomogram model (**Figures 4E, F**).

### Expression of Claudin 18 protein in colorectal cancer tissues and its correlation with clinical pathological parameters

**Figure 5:**
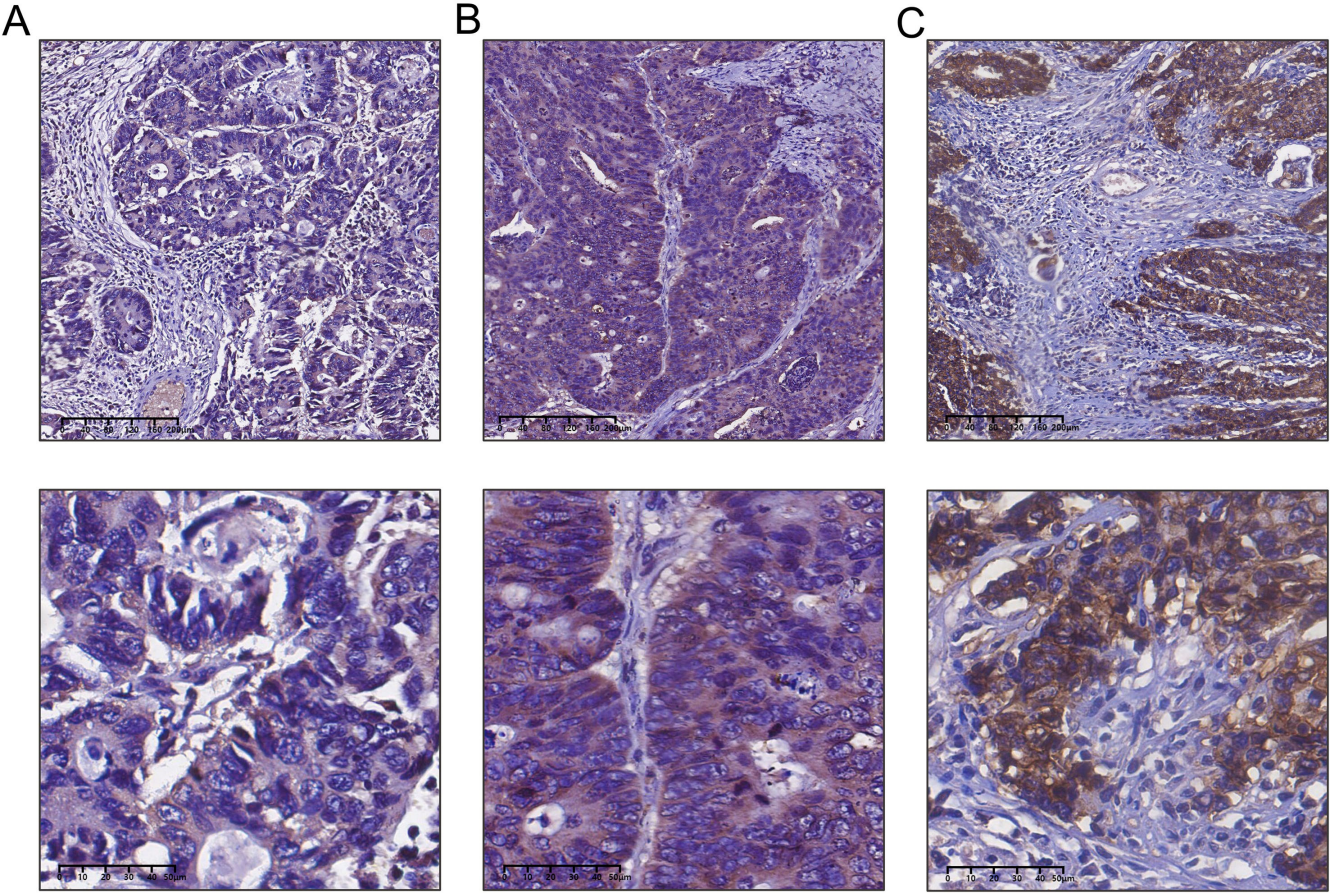
Detection of claudin 18 protein expression in colorectal cancer tissues by immunohistochemistry. **(A)** Weak expression of Claudin 18 protein in colorectal cancer; **(B)** Moderate expression of Claudin 18 protein in colorectal cancer; **(C)** Strong expression of Claudin 18 protein in colorectal cancer.

We performed an immunohistochemical analysis to evaluate Claudin 18 protein expression levels in CRC tissues. The results showed that Claudin 18 protein primarily localized to the cell membrane and cytoplasm (**Figure 5**). In colorectal cancer tissues with PNI, the positive expression rate of Claudin 18 was 56.8% (50/88), while it was 33.3% (10/30) in tissues without PNI. Statistical analysis indicated a significant difference in Claudin 18 protein expression levels between the two groups (P < 0.05, (**Table 1**). We further analyzed the correlation between Claudin 18 protein expression in colorectal cancer tissues and clinical pathological parameters. The results revealed that Claudin 18 protein expression levels were significantly associated with patient age and lymphatic invasion status (P < 0.05, **Table 2**).

**Table 1:** Expression of Claudin 18 protein in colorectal cancer tissues with and without perineural invasion.

PNI status	Number of positive cases (%)	Number of negative cases (%)	Total number of cases	*P* value
PNI	50 (56.8%)	38 (43.2%)	88	0.0263*
without PNI	10 (33.3%)	20 (66.7%)	30	

*Chi-Square Test.

**Table 2:** Relationship between Claudin 18 protein expression in colorectal cancer tissues and clinical pathological parameters.

Characteristic	Category	Positive cases, n (%)	Negative cases, n (%)	Total, n	*P* value
Gender	Female	23 (19.5)	30 (25.4)	53	0.144
	Male	37 (31.4)	28 (23.7)	65	
Age	<60	9 (7.6)	20 (16.9)	29	0.014
	≥60	51 (43.2)	38 (32.2)	89	
T stage	T1	2 (1.7)	1 (0.8)	3	0.399
	T2	3 (2.5)	8 (6.8)	11	
	T3	20 (16.9)	17 (14.4)	37	
	T4	35 (29.7)	32 (27.1)	67	
N stage	N0	25 (21.2)	25 (21.2)	50	0.875
	N+	35 (29.7)	33 (28.0)	68	
M stage	M0	58 (49.2)	56 (47.5)	114	1
	M1	2 (1.7)	2 (1.7)	4	
Histological type	Colon cancer	32 (27.1)	31 (26.3)	63	0.99
	Rectal cancer	28 (23.7)	27 (22.9)	55	
Differentiation	High	16 (13.6)	18 (15.3)	34	0.344
	Moderate	42 (35.6)	40 (33.9)	82	
	Low	2 (1.7)	0 (0.0)	2	
MMR status	pMMR	38 (32.2)	28 (23.7)	66	0.1
	dMMR	22 (18.6)	30 (25.4)	52	
Lymphatic invasion*	No	14 (12.0)	24 (20.5)	38	0.042
	Yes	45 (38.5)	34 (29.1)	79	
Total		60 (50.8)	58 (49.2)	118	

*Data on lymphatic invasion status was unavailable for one patient.

## Discussion

CRC exhibits heterogeneity and complex carcinogenic mechanisms, leading to significant variability in prognostic outcomes, even among cases with similar pathological and histological features. Recent research has shown a strong link between PNI and poor prognoses in CRC patients (11, 12). PNI is significantly associated with lower survival rates and higher recurrence rates in CRC patients. It is also recognized as a critical pathway for metastasis, along with lymphatic and hematogenous spread (13, 14). Additionally, the presence of PNI indicates poorer clinical outcomes (15). The NCCN clinical practice guidelines classify PNI as a high-risk factor for CRC recurrence, recommending adjuvant therapy for stage II CRC patients with positive PNI status (4). PNI frequently occurs in various types of cancer. Therapeutic strategies for CRC have diversified to include surgery, radiotherapy, chemotherapy, targeted therapy, and immunotherapy. However, long-term survival rates for patients remain low (16). Given these challenges, it is essential to develop effective prognostic models for predicting outcomes. These models aim to enhance the prediction of patient outcomes and enable timely interventions and personalized treatments. This study investigates molecular markers associated with perineural invasion in colorectal cancer. It aims to combine these findings with clinical characteristics to develop a nomogram model for predicting mortality risk in patients with stage II CRC.

This study analyzed transcriptomic and clinical data related to CRC from the TCGA database. We identified 33 genes that are differentially expressed in relation to PNI and stage II CRC. The survival analysis subsequently revealed five genes that are significantly correlated with overall survival: CLDN18, FTCD, MSH4, MUC6, and PLG. Notably, high levels of CLDN18 and FTCD were confirmed as independent prognostic factors for poor outcomes in patients with stage II CRC. While constructing the prognostic model, we chose CLDN18 and FTCD as primary biomarkers and combined them with clinical-pathological parameters, including age and sex. This comprehensive Nomogram model not only accounts for the influence of these biomarkers but also incorporates clinical characteristics to prevent the omission of valuable variables.

CLDN18, a member of the Claudins protein family, is a key component of tight junctions (TJs) that regulate molecular flow between cells by forming a paracellular barrier (17, 18). The CLDN18 gene has two different exon 1 sequences. Alternative splicing after transcription produces two protein isoforms, CLDN18.1 and CLDN18.2, which differ only in their N-terminal sequences. Research on CLDN18 has made significant advancements, particularly in relation to gastric cancer. When gastric cancer is classified into four molecular subtypes, the fusion of CLDN18 with ARHGAP can be observed specifically in the genomically stable subtype (19, 20). Due to its specific expression in gastric cancer, CLDN18 has emerged as a potential therapeutic target. An antibody that targets CLDN18.2 has been successfully developed and used in gastric cancer treatment (21). Existing studies indicate that normal colonic tissue does not express CLDN18 (22). IHC assessments of CLDN18 expression across all subtypes of CRC have shown significant variability, with positive expression rates ranging between 15% and 42% (23–25). Particularly in the serrated adenocarcinoma subtype, CLDN18 expression has been closely linked to an increase in lymph node metastases and more advanced overall staging (26). However, current reports on CLDN18 expression in various types of polyps do not yet agree (26, 27). In CRC patients, the protein expression level of CLDN18 has been identified as an independent predictor of overall survival (28). This study is the first to investigate the impact of CLDN18 mRNA expression levels on the overall survival of patients with stage II CRC, revealing that high expression of CLDN18 constitutes an independent factor for poor prognosis in this patient population. Additionally, this study employed immunohistochemical techniques to assess the expression levels of Claudin 18 protein in colorectal cancer tissues. The results demonstrated that Claudin 18 protein expression was significantly elevated in tissues exhibiting perineural invasion and was closely associated with clinical-pathological parameters such as age and lymphatic invasion. This finding not only enhances the credibility of Claudin 18 as a prognostic marker for CRC but also provides empirical evidence for its role in the mechanisms of invasion and metastasis.

Formimidoyltransferase cyclodeaminase (FTCD) is a bifunctional enzyme that plays a critical role in the catabolism of histidine. Additionally, FTCD collaborates with proteins p97 and p47 during mitosis to perform membrane tethering functions. Existing research indicates that abnormal FTCD expression is linked to various disease states. These include autoimmune hepatitis, disruptions in histidine and folate metabolism, and abnormalities in arsenic metabolism (29–31). However, studies on FTCD in the context of cancer are limited, primarily focusing on hepatocellular carcinoma (HCC), with little evidence reported for other tumor types. In HCC, FTCD significantly contributes to disease progression. Specifically, knocking out FTCD in liver cells promotes chronic liver cancer induced by diethylnitrosamine and spontaneous liver tumors in mice (32). Moreover, the downregulation of FTCD expression in the HCC has been correlated with lipid metabolic abnormalities and poor patient prognosis (32). Notably, FTCD stimulates macrophages to polarize towards the M1 phenotype. This action inhibits the proliferation of HCC cells (33). Furthermore, FTCD has been shown to positively influence the suppression of HCC through the regulation of apoptosis, DNA damage repair, and the phosphatidylinositol 3-kinase (PI3K)/Akt signaling pathway (34).With regard to prognosis, a decrease in FTCD expression has been positively correlated with favorable outcomes in HCC (35, 36). Further investigations have revealed that the downregulation of FTCD can diminish the activity of HIF-1α under hypoxic conditions, thereby increasing the sensitivity of HepG2 cells (37). This study is the first to explore the prognostic value of FTCD in CRC. Our findings indicate that high expression levels of FTCD are significantly associated with poor prognosis in patients with stage II CRC.

## Conclusions

This study assessed the accuracy of the Nomogram model in predicting outcomes for patients with stage II CRC using calibration curves, AUC values, and DCA curves. Unlike approaches that rely solely on individual biomarkers or clinical characteristics, this model incorporates multiple factors, thereby enhancing its predictive capability. While our results are promising, we must acknowledge several limitations in our research. Primarily, our analysis focused on the genes CLDN18 and FTCD. However, the development and progression of CRC is a complex process that involves multiple genes and signaling pathways. Hence, in future investigations, we plan to utilize an external validation cohort to confirm our findings and assess the model’s validity. Additionally, future studies should aim to explore other genes and molecular markers associated with colorectal cancer prognosis, with the objective of providing more comprehensive and precise prognostic assessments for patients.

## Data Availability

The datasets presented in this article are not readily available because The dataset used in this study was obtained from The Cancer Genome Atlas (TCGA) database, which is publicly available. The data is de-identified and available for academic research purposes. Access to the dataset is governed by TCGA’s data access policies, ensuring that the data is used in accordance with ethical standards and patient confidentiality requirements. Data usage for commercial purposes is restricted. Requests to access the datasets should be directed to CC, changchen9185@163.com.
